# Diagnostic utility of *DREAM* gene mRNA levels in thyroid tumours

**DOI:** 10.20945/2359-3997000000028

**Published:** 2018-03-23

**Authors:** Fernando A. Batista, Marjory A. Marcello, Mariana B. Martins, Karina C. Peres, Ulieme O. Cardoso, Aline C. D. N. Silva, Natassia E. Bufalo, Fernando A. Soares, Márcio J. da Silva, Lígia V. Assumpção, Laura S. Ward

**Affiliations:** 1 Universidade Estadual de Campinas Universidade Estadual de Campinas Faculdade de Ciências Médicas Laboratório de Genética Molecular do Câncer Campinas SP Brasil Laboratório de Genética Molecular do Câncer (Gemoca), Faculdade de Ciências Médicas (FCM), Universidade Estadual de Campinas (Unicamp), Campinas, SP, Brasil; 2 Hospital A. C. Camargo Hospital A.C. Camargo – Fundação Antonio Prudente Departamento de Patologia São Paulo SP Brasil Departamento de Patologia, Hospital A.C. Camargo – Fundação Antonio Prudente, São Paulo, SP, Brasil; 3 Universidade Estadual de Campinas Universidade Estadual de Campinas Centro de Biologia Molecular e Engenharia Genética Campinas SP Brasil Centro de Biologia Molecular e Engenharia Genética (CBMEG), Universidade Estadual de Campinas (Unicamp), Campinas, SP, Brasil

**Keywords:** Calsenilin, diagnostic, follicular thyroid lesion, KChIP-3, thyroid nodules

## Abstract

**Objective:**

The transcriptional repressor DREAM is involved in thyroid-specific gene expression, thyroid enlargement and nodular development, but its clinical utility is still uncertain. In this study we aimed to investigate whether *DREAM* mRNA levels differ in different thyroid tumors and how this possible difference would allow the use of *DREAM* gene expression as molecular marker for diagnostic and/or prognosis purpose.

**Materials and methods:**

We quantified *DREAM* gene mRNA levels and investigated its mutational status, relating its expression and genetic changes to diagnostic and prognostic features of 200 thyroid tumors, being 101 malignant [99 papillary thyroid carcinomas (PTC) and 2 anaplastic thyroid carcinomas] and 99 benign thyroid lesions [49 goiter and 50 follicular adenomas (FA)].

**Results:**

Levels of mRNA of *DREAM* gene were higher in benign (0.7909 ± 0.6274 AU) than in malignant (0.3373 ± 0.6274 AU) thyroid lesions (p < 0.0001). *DREAM* gene expression was able to identify malignancy with 66.7% sensitivity, 85.4% specificity, 84.2% positive predictive value (PPV), 68.7% negative predictive value (NPV), and 75.3% accuracy. *DREAM* mRNA levels were also useful distinguishing the follicular lesions FA and FVPTC with 70.2% sensitivity, 73.5% specificity, 78.5% PPV, 64.1% NPV, and 71.6% accuracy. However, *DREAM* gene expression was neither associated with clinical features of tumor aggressiveness, nor with recurrence or survival. Six different genetic changes in non-coding regions of *DREAM* gene were also found, not related to *DREAM* gene expression or tumor features.

**Conclusion:**

We suggest that *DREAM* gene expression may help diagnose thyroid nodules, identifying malignancy and characterizing follicular-patterned thyroid lesions; however, it is not useful as a prognostic marker.

## INTRODUCTION

An important increase in the incidence of thyroid cancer has been observed worldwide, mainly because of a better access to high resolution image exams, such as ultrasound, and frequent fine-needle aspiration (FNA) biopsies of small nodules (1–4). Despite the advantages of cytological analysis using FNA, this method has limitations, not being able to diagnose reliably a substantial part of thyroid nodules. Hence, many patients are submitted to thyroid resection for a definitive diagnosis, and postsurgical exams reveal no need for surgery in approximately 75% of these cases (4–7). A series of diagnostic markers has been proposed and some molecular platforms are particularly helpful in thyroid cancer diagnosis, but their implementation in clinical routine is still difficult (8). The determination of prognosis in thyroid cancer patient is problematic as well. Although most patients have satisfactory outcome, recurrences are relatively frequent and up to 10-15% of cases may present an aggressive behavior characterized by metastases during follow-up, eventually leading to death (9). Reliable markers able to predict poor outcome during followup are utmost needed in clinical practice (2,4).

The *DREAM* gene (downstream regulatory element antagonist modulator) has been mapped to chromosome 2q11.1 and has nine exons. The product of this gene, an homonymous protein, also identified as KchIP-3 (K channel-interacting protein 3) or calsenilin, belongs to the KchIP family, composed by four proteins (KChIP1-4) (10). DREAM has the ability to directly bind to specific DNA sites, the downstream regulatory elements (DRE), acting as a transcriptional repressor (11). Under basal conditions, DREAM binds to DRE, resulting in repression of transcription of target genes. Activation of DREAM, which can happen by increasing nuclear calcium or by direct phosphorylation by PKA, results in the disruption of the DREAM-DRE binding, which enables gene transcription (10,12,13). DREAM may also connect to Kv4 potassium channels in the membrane as an accessory subunit, regulating their opening through stimulation by calcium (14). *DREAM* expression is found in the central nervous system, organs of the immune system, tests and thyroid (10). Although previous studies have suggested that DREAM has an important role as a mediator in several thyroid signaling pathways, and it is also involved in the regulation of follicular cellular functions (15–17), a possible clinical utility of DREAM as a biomarker for diagnosis or prognosis of thyroid lesions has not been explored. For these reasons, we quantified *DREAM* mRNA levels, and investigated the occurrence of its genetic alterations both in benign and malignant thyroid nodules, comparing the presence of these alterations and/or expression levels to diagnostic and prognostic features of these tumors.

## MATERIALS AND METHODS

### Patients

We investigated a total of 200 patients whose tissue samples were maintained in the tissue bank of the AC Camargo Cancer Hospital, São Paulo, Brazil. Fresh thyroid tissue samples were obtained from 101 patients diagnosed with thyroid carcinoma (TC): 99 papillary thyroid carcinomas (PTCs), including 63 classic papillary thyroid carcinomas (CPTC), 36 follicular variants of papillary thyroid carcinomas (FVPTC); and two anaplastic thyroid carcinomas (ATC). In addition, there were 49 nodular goiters and 50 follicular adenomas (FA). Four normal tissues from the contralateral lobe of four goiter cases were obtained as well. The diagnosis of TC was based on standard clinical criteria described in our previous article (18).

All patients were treated according to a standard protocol (19,20) and followed for 12-87 months with a median follow-up of 3.16 years (38 ± 17 months).

This study was approved by the Research Ethics Committees of the institutions involved (Process # 821,805). All procedures performed in this study were in accordance with the ethical standards of the institutional and national research committee and the 1964 Helsinki declaration and its later amendments or comparable ethical standards.

### Quantitative real-time PCR (qPCR)

We were able to extract RNA from all 204 samples. Total RNA was extracted from pulverized frozen thyroid tissues using Trizol reagent (Invitrogen Life Technologies Inc., Carlsbad, CA, USA), according to the manufacturer instructions. The samples were digested with Amplification Grade DNAse I (Life Technologies, Rockville, MD, USA) and reversetranscribed using SuperScript III reverse-transcriptase (Invitrogen Life Technologies Inc.). The assays were carried out with the use of commercially available TaqMan gene expression assays (Applied Biosystems) for *DREAM* (Hs01106289_m1) relative to the internal reference gene *GAPDH* (Hs02758991_g1). Reactions were prepared with TaqMan gene expression master mix (Applied Biosystems), according to the manufacturer protocol. Analysis was performed with a 7500 RT-PCR system (Applied Biosystems, Foster City, CA, USA), using a four-stage program: 50°C for 2 min, 95°C for 10 min, 40 cycles of 95°C for 15s, and 60°C for 1 min. Each sample was assayed in triplicate. Threshold cycle (Ct) was obtained using the sequence detection software (Applied Biosystems SDS v1.3 Software). We used ΔΔCT, in which the amount of target (*DREAM*) normalized to an endogenous reference and relative to calibrator is given by 2-^ΔΔT^. The expression levels were given in arbitrary units (AU).

### Sequencing the coding regions of *DREAM* gene

The coding regions of *DREAM* gene were amplified by PCR using the following primers (all 5′–3′, forward and reverse respectively): AGGGGTGGAGCGATAGAAG and CAAAGGAAAGTGGAACAAGAG for exon 1; CCCATCTTACACCATAGCCA and GGGAAGGGTGTGAATGAATG for exon 2; GGTAGTCATGCAAAGAGAGTTC and TTTCCCACAACACATAAGCC for exon 3; CAAGGGGGTGGAGAGAGG and CCCAGGGTGACTCACAAGAT for exons 4 and 5; AATGGATGCCGTCAGTCTCT and CCGAGAACACTTGCTGAGCT for exon 6; CTTCTCTCTCCAGCTCGTC and GAGTAGGGAGGCTCAGAGG for exon 7; CCAGAGTAGTCACAGGGGCA and AGACAAGAGGGCAAGTGGAG for exon 8; CTCCCTGCACCAATAAGAC and CTGGCAGGATGGAGGTTTCT for exon 9. PCR was performed in 20 μL volume of a mixture containing 100 ng of DNA, 5 mM of each primer, 2 μL of 10X PCR Buffer, 150 μM of each dinucleotide triphosphate, 1U Taq DNA polymerase, 1.5 mM MgCl_2_ and deionized water up to 20 μL. Amplifications were carried out for 35 cycles of 94°C for 30 seconds, 55°C for 50 seconds and 72°C for 1 minute, with an initial denaturation step of 94°C for 5 minutes and a final extension step of 72°C for 10 minutes, using a MJ PTC-200 PCR system. Purification was performed using ExoSAPIT (USB Products, Cleveland, OH). The samples were submitted to automated sequencing using the SANGER method. For this reaction, we used for each sample: 13.5 μL of water, 4 μL of save money 5x buffer, 1 μL of each primer, 0.5 μL of BigDye buffer and 1 μL of the purified sample. The following cycles were used in the sequencing reaction: initial cycle of 1 min and 30s at 96°C, followed by 25 cycles of 96°C for 12s, 50°C for 6s and 60°C for 4 min. Sequencing reactions were performed using Eppendorf PCR BD-3700 system and separated on an ABI Prism 3700 DNA Analyzer (Applied Biosystems, Foster, CA, USA). All sequences were analyzed using CLC DNA Workbench^®^ (Katrinebjerg, Denmark) software and compared with the *DREAM* genomic sequence (ENSG00000115041).

### Statistical analysis

Statistical analysis was carried out using the SAS System for Windows (Statistical Analysis System, version 9.1.3, Service Pack 3 Institute Inc., 2002–2003, Cary, NC, USA). For the analysis of correlation of *DREAM* gene expression and clinical and pathological features of aggressiveness, patients were classified according to age of diagnosis, gender, tumor size, presence of extrathyroidal invasion, presence of capsule, multifocality, presence of metastasis at diagnosis and TNM. For the comparison with patients outcome, the population as classified according to response to treatment, as recommended by the latest ATA guidelines (4). The Mann–Whitney tests were used to compare continuous or arranged measures between two groups; Kruskal–Wallis test was used to compare three or more groups. The accuracy of gene expression studies to predict malignancy and/or differentiate follicular lesions was evaluated using receiver operating curve (ROC) analysis, based on predicted probabilities from logistic regression models. Recurrence-free survival was calculated using Kaplan–Meier survival curves with log rank comparison. For this analysis, the TC patients were divided in two groups, low expression of *DREAM* (≤ 0.2AU) and normal/hyperexpression of *DREAM* (> 0.2AU). All tests were conducted at the significance level *p* = 0.05.

## RESULTS

As expected, thyroid cancer patients were predominantly females (75.3%) aged 14–70 years old (41.1 ± 14.3 years) at the time of diagnosis. The 101 patients of the thyroid cancer group did not differ from the 99 individuals with benign thyroid diseases concerning gender (67 females and 22 males *vs.* 78 females and 16 males, respectively) or age at diagnosis (45.6 ± 16.3 years old *vs.* 48.5 ± 14.8 years old, respectively). Multifocality was observed in 37% of the patients and 26% presented invasion of the capsule. Stage I (66%) and stage II (29%) were more frequent than stage III (3%) and stage IV (2%) cases. Metastasis at the time of diagnosis was observed in 45% of the patients.

### *DREAM* mRNA levels

**Figure 1:**
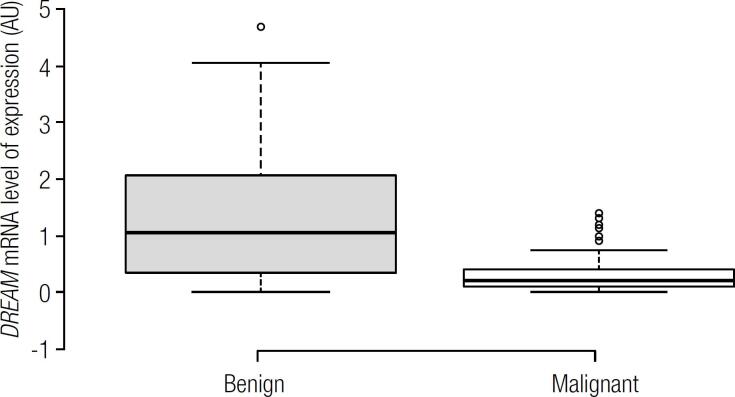
*DREAM* mRNA expression by quantitative PCR in benign and malignant thyroid lesions (*P* < 0.0001).

**Figure 2:**
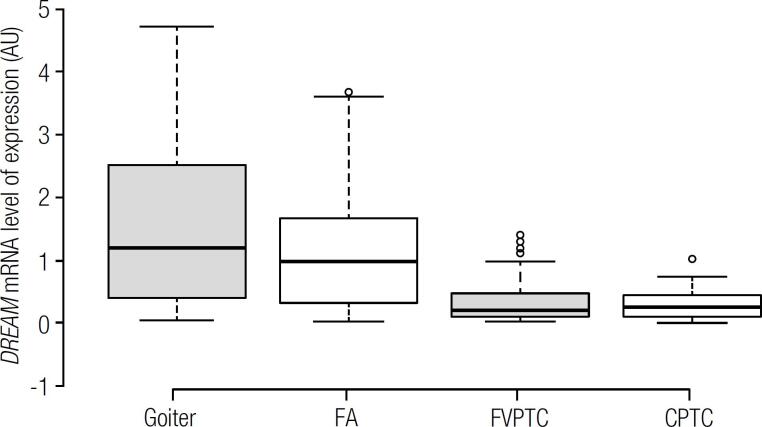
*DREAM* mRNA expression by quantitative PCR in goiter, follicular adenoma (FA), follicular variant papillary thyroid carcinoma (FVPTC) and classic papillary thyroid carcinoma (CPTC).

*DREAM* mRNA level comparison between benign and malignant lesions is summarized in Table 1. As presented in Figure 1, the levels were significantly higher in benign (0.7909 ± 0.6274 AU) than in malignant nodules (0.3373 ± 0.6274 AU; p < 0.0001). Among the different histological types of tumors, we found higher mRNA levels of *DREAM* gene in goiter, followed by FA, FVPTC and CPTC respectively, as represented in Figure 2. Furthermore, *DREAM* gene expression was able to distinguish goiter and FA, goiter and FVPTC, goiter and CPTC, FA and CPTC, and the follicular-patterned lesions, FA and FVPTC, as shown in Table 1.

**Table 1:** Comparisons of different histopathological types according to their *DREAM* mRNA levels

Analyzed groups	*DREAM* mRNA levels ΔΔCt (qPCR)	*p* value
Malignant *vs*. Benign	0.3373 ± 0.6274 *vs*. 0.7909 ± 0.6274	< 0.0001
Goiter *vs*. FA	0.7816 ± 0.6512 *vs*. 0.8001 ± 0.6160	0.6725
Goiter *vs*. FVPTC	0.7816 ± 0.6512 *vs*. 0.3449 ± 0.2221	0.0069
Goiter *vs*. CPTC	0.7816 ± 0.6512 *vs*. 0.3281 ± 0.2261	0.0002
FA *vs*. FVPTC	0.8001 ± 0.6160 *vs*. 0.3449 ± 0.2221	0.0002
FA *vs*. CPTC	0.8001 ± 0.6160 *vs*. 0.3281 ± 0.2261	< 0.0001
FVPTC *vs*. CPTC	0.3449 ± 0.2221 *vs*. 0.3281 ± 0.2261	0.6378

Aiming to investigate the diagnostic utility of *DREAM* gene expression, we further performed ROC analysis based on predicted probabilities from logistic regression models. Using a cutoff of 0.528 AU, *DREAM* was able to predict malignancy in thyroid nodules with 66.7% sensitivity, 85.4% specificity, 84.2% positive predictive value (PPV), 68.7% negative predictive value (NPV), and 75.3% accuracy. *DREAM* mRNA levels were also useful in distinguishing the follicular lesions FA and FVPTC with 70.2% sensitivity, 73.5% specificity, 78.5% PPV, 64.1% NPV, and 71.6% accuracy, using a cutoff value of 0.405 AU.

**Figure 3:**
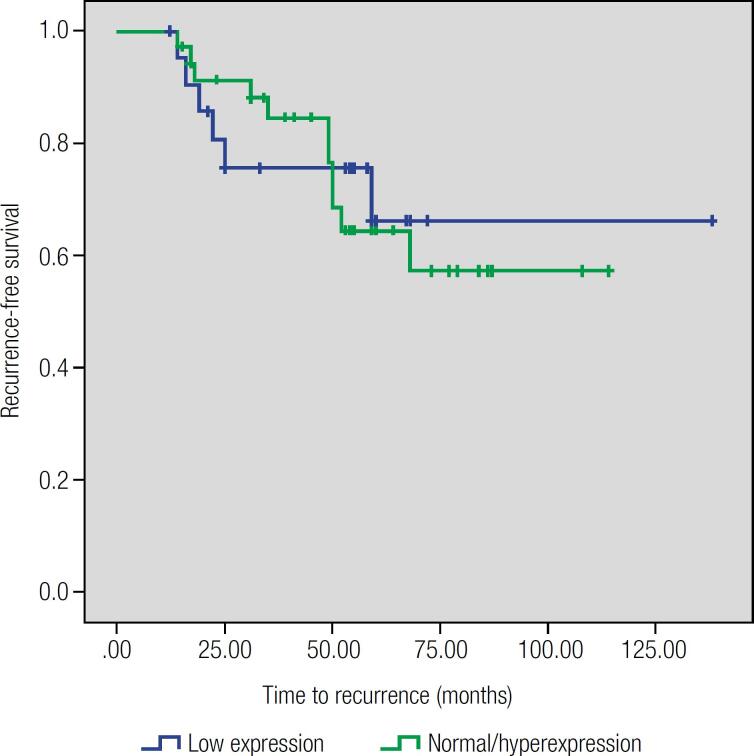
Kaplan-Meier curve comparing patterns of recurrence-free survival between patients according to DREAM gene expression.

Although DREAM gene mRNA levels were different among thyroid lesions, there was no association with any clinical or pathological parameter of tumor aggressiveness and there was no association with patient outcomes (Table 2, Figure 3).

**Table 2:** Comparison of DREAM mRNA levels to clinical and pathological features of aggressiveness and outcome of 101 thyroid carcinomas

Variable		*DREAM* expression	*p* value
Age of diagnosis			
	< 45	0.44 ± 0.51	0.76
	> 45	0.46 ± 0.66	
Gender			
	Male	0.34 ± 0.30	0.9
	Female	0.48 ± 0.67	
Tumour size			
	< 2 cm	0.20 ± 0.31	
	2 – 4 cm	0.33 ± 0.33	0.23
	> 4 cm	0.39 ± 0.45	
Extrathyroidal invasion			
	Present	0.46 ± 0.65	0.83
	Absent	0.40 ± 0.45	
Presence of capsule			
	Present	0.48 ± 0.60	0.27
	Absent	0.44 ± 0.61	
Multifocality			
	Present	0.35 ± 0.26	0.24
	Absent	0.49 ± 0.72	
Metastasis at diagnosis			
	Present	0.48 ± 0.62	0.32
	Absent	0.38 ± 0.48	
TNM			
	I	0.94 ± 0.42	0.43
	II	0.71 ± 0.38	
	III	0.67 ± 0.57	
	IV	0.41 ± 0.00	
Outcome*			
	Excellent response	0.49 ± 0.10	0.61
	Incomplete response	0.82 ± 0.40	

* Classification according to recommendation of American Thyroid Association 2015 guidelines (4). Incomplete responses were grouped together due to insufficient number of structural incomplete response cases for statistical analysis. Indeterminate response cases were excluded from the analysis.

### DREAM mutational analysis

We were able to perform mutational analysis of all 9 exons of *DREAM* gene in all 200 samples. There were no genetic changes in the coding regions. However, we found 6 single base substitutions in noncoding regions near the amplified coding regions (Table 3). No association was found regarding clinical or pathological features, and we were unable to demonstrate any variation of mRNA levels in association with the observed sequence substitutions.

**Table 3:** Genetic changes in *DREAM* gene found in this study

ID (rs)	Localization	Nucleotide	Allelic frequency
–	Promoter region	IVS-95T>A	A = 0.9975 (399/400) T = 0.0025 (01/400)
rs2248415	Intron1	IVS1+41C>G	C = 0.2500 (100/400) G = 0.7500 (300/400)
–	Intron1	IVS2-131C>T	C = 0.0050 (02/400) T = 0.9950 (398/400)
rs117109173	Intron3	IVS3+10G>C	C = 0.940 (376/400) G = 0.0600 (24/400)
–	Intron3	IVS3+17T>C	T = (0.0025 (01/400) C = 0.9975 (399/400)
rs58372613	Intron7	IVS8-118C>T	C = 0.4300 (112/400) T = 0.5700 (288/400)

## DISCUSSION

In the present study we report higher mRNA levels of *DREAM* gene in benign thyroid tumours when compared to malignant thyroid lesions. By comparing different histologic types of tumours, we also described higher *DREAM* gene expression not only presents potential identifying malignancy but also distinguishing the follicular-patterned lesions AF and CPVF. We also described the presence of intronic genetic changes of *DREAM* gene in thyroid nodules patients. Although this is, to our knowledge, the first study of the *DREAM* gene comprehending malignant and benign thyroid nodules, previous studies have already demonstrated the importance of DREAM for the normal function of the thyroid gland, which may justify the possible loss of expression during the dedifferentiation process of the gland. Rivas and cols., demonstrated that, in thyroid follicular cells, DREAM interacts directly with the thyroid transcription factor-1 (TTF-1) by modulating its transcriptional activity, and acts in the regulation of gene expression of thyroglobulin and the other two main thyroid-specific transcription factors PAX8 and FOXE1 (15), both involved in thyroid differentiation (21,22). DREAM can still act as intracellular effector of TSH receptor (TSHR), activating the signaling cascade by cAMP (adenosine 3′,5′-cyclicmonophosphate) independently of stimulation by TSH, which is suggested by Rivas and cols., as a possible mechanism of development of multinodular goiter by describing overexpression of DREAM in 10 of 16 human samples (16). Similarly, Shinzato and cols. described overexpression of *DREAM* gene in 53.3% of a cohort composed of 60 multinodular goiter patients (23). Our data is compatible with these findings, being goiter the lesion which presented higher levels of *DREAM* mRNA compared to any other histological type of the analyzed thyroid samples. The same study also reports mutational analysis of *DREAM*. The SNPs rs2248415 and rs117109173, found in a 75 and 6% of our patients respectively were also described by them in 91.6 and 8.3% of their patients respectively. In our analysis, the intronic polymorphisms did not correlate in *DREAM* mRNA levels in different thyroid lesions and, similarly to previously published, do not seem to be associated with differential *DREAM* expression (23).

The expression pattern of *DREAM* in different thyroid lesions rise the hypothesis of a possible involvement of this transcription repressor in the tumorigenesis and dedifferentiation of the thyroid gland. Although the exact mechanism by which DREAM could possibly play a role in this process remains unknown, there are multiple paths to be explored. The lower expression of *DREAM* in malignant lesions may play a role in thyroid carcinogenesis given to the proapoptotic activity, thanks to the direct interaction with the presenilin 2(PS2) protein, which culminates in the activation of the caspase cascade (24). The above mentioned transcriptional modulation activity of DREAM on *PAX8* and *FOXE1* gene expression (15) could also explain the lower mRNA levels in malignant thyroid tissue, since both genes are involved in thyroid differentiation. Our previous findings about mRNA levels of thyroid specific transcription factors in thyroid tumours corroborate this hypothesis: both *PAX8* and *FOXE1* follow a similar expression pattern than *DREAM*, being highly expressed in benignant lesions compared to malignant lesions (18). A third possibility would be the involvement of DREAM as repressor of the immune response. Savignac and cols. have previously demonstrated that transient knockdown of *DREAM* induced basal expression of interleukin-2 and interferon gamma in wild-type splenocytes (25). Recent publications from our group have demonstrated the important role which the immune system plays in thyroid cancer behaviour (26–29). Further investigation could lead to the identification of DREAM as one of the many molecules which take part in this association.

The differential expression of the *DREAM* gene described by us rises the possibility of use of this gene as a molecular marker. Although *DREAM* mRNA levels failed to identify aggressiveness and predict prognosis, there is potential for the use as a diagnostic marker. A comparison with some of the currently available panels of molecular markers can be seen on Table 4. *DREAM* specificity (85%), was better than Afirma (52%), Rosetta microRNA classifier (72%) and ThyGenX^®^/ ThyraMIR™ (85%). Despite its low NPV (69%), *DREAM* presented the second best PPV (84%), higher than other four panels (ThyroSeq v2, 77%; Afirma, 47%; Rosetta microRNA classifier, 59%; and ThyGenX^®^/ ThyraMIR™, 74%). Perhaps the most interesting comparison is the one between *DREAM* expression and Afirma, given the fact that this gene expression classifier (GEC) test which possesses high NPV and sensitivity (93 and 92% respectively), has relatively low specificity and PPV (52 and 47% respectively). As a result, more than a half of the patients tested positive by the Afirma GEC may still have a benign disease on surgical pathology. On the contrary, *DREAM* presented much higher PPV and specificity values (84 and 85% respectively) but much lower NPV and sensitivity value (69 and 67% respectively). We suggest that *DREAM* gene expression may not be useful as a single marker, but could be part of a panel of markers of malignancy, like the GEC test. Also, it may help identify follicular lesions, one of the major challenges in clinical practice.

**Table 4:** Comparison of sensitivity, specificity, NPV and PPV values of *DREAM* mRNA levels in thyroid tumours with some currently available panels of thyroid cancer molecular markers

	Sensitivity	Specificity	NPV	PPV
*DREAM* mRNA levels	67%	85%	69%	84%
Seven-gene panel (30)	63%	99%	94%	88%
ThyroSeq v2^®^ (31)	91%	92%	97%	77%
Afirma (32)	92%	52%	93%	47%
Rosetta microRNA classifier™ (33)	85%	72%	91%	59%
ThyGenX^®^ and ThyraMIR™ (34)	89%	85%	94%	74%

In conclusion, our investigation demonstrated a possible diagnostic utility of *DREAM* gene mRNA levels in the identification of thyroid nodule malignancy and differentiation of follicular-patterned thyroid lesions. In addition, we demonstrated the presence of intronic changes of *DREAM* gene in patients with thyroid nodules, although these changes were not related to clinical features.
